# Study on dynamic wetting and synergistic effect of surfactants on bituminous coal surface in coal mines

**DOI:** 10.1371/journal.pone.0334367

**Published:** 2026-02-05

**Authors:** Jiangpeng Ju, Ju Zhao

**Affiliations:** 1 School of Chemical and Energy Engineering, Shaanxi Energy Institute, Xianyang, China; 2 Shaanxi Key Laboratory of Higher Education Institutions for Intelligent Prevention and Control of Coal Mine Disasters, Xianyang, China; Henan Polytechnic University, CHINA

## Abstract

With the continuous advancement of green mining initiatives in China, dust control during coal mining has become a critical issue for occupational health and ecological environmental safety. In response to the issues of coal dust pollution generated during coal mining and the inefficiencies of water spray dust suppression, this study investigates the fundamental mechanism by which multi-component surfactants synergistically form a stable adsorption layer on bituminous coal surfaces. The research employs macro-scale surface tension measurements, dynamic contact angle analysis, XPS spectroscopy, SEM for microstructural morphology, and molecular dynamics simulations. The results identify the optimal formulation as a 1:1 volume ratio mixture of 0.2% SDS and 0.1% CDEA. The synergistic effect of hydrogen bonding and electrostatic interactions first promotes the formation of a complex between the sulfate group of SDS and the amide group of CDEA. This complex then packs more efficiently, leading to an increased molecular density at the interface. The surface tension of the system is reduced to 35.23 mN/m, while the adhesion work and immersion work of the solution on the bituminous coal surface reach their maximum values. The spreading work is –0.48 mN/m, closest to the threshold of spontaneous spreading. This research offers theoretical support for developing a new generation of high-performance and environmentally friendly dust suppressants.

## 1. Introduction

With the advancement in mechanization and automation of coal mining, dust generation at the working face has significantly increased. This dust not only threatens the occupational health of workers but also severely impacts ambient air quality and mine safety production [1–3]. According to the National Health Commission of China, by the end of 2022, there were 923,000 reported cases of occupational pneumoconiosis in China, accounting for approximately 88.9% of all occupational disease cases. Currently, mine dust has become the primary cause of occupational health hazards among miners. Pneumoconiosis is an incurable disease with no medical endpoint. The average annual medical cost per pneumoconiosis patient in China is ¥19,050, with additional expenses—including transportation, nutrition, and lost wages—amounting to ¥45,790 per year. The average lifetime economic burden per patient is estimated to be ¥2.075 million [4,5]. Therefore, developing efficient and cost-effective dust control technologies, particularly during coal transportation and storage, is of significant importance for improving environmental quality, reducing the incidence of occupational diseases, and alleviating economic burdens [6].

Spray dust suppression is a commonly used and highly efficient dust pollution control technology in coal mines. However, due to the low surface energy and non-polar characteristics of coal surfaces, which result in high interfacial tension, liquid droplets struggle to adequately wet and spread on coal dust surfaces, significantly limiting the dust removal efficiency [7–9]. Surfactants possess the ability to markedly reduce the surface tension of solutions, thereby enhancing the wetting and penetration capability of the solution towards dust, promoting its spreading and adsorption on coal dust surfaces, and improving dust agglomeration and settlement [10,11]. By optimizing the type and blending ratio of surfactants, more efficient and durable dust control can be achieved under complex working conditions [12–14].

There is a wide variety of surfactants, and differences in their molecular structures directly determine the wetting kinetics and thermodynamic processes at the water–coal interface. Numerous scholars have investigated the wetting performance of different types of surfactants [15–17]. Meng et al. [18] demonstrated that the number of hydrophilic groups in a surfactant is positively correlated with its wetting performance. Wang et al. [19] found that compounded solutions of imidazole-based ionic liquids and surfactants exhibit significant synergistic effects at specific ratios, with [Bmim][Cl] showing a particularly notable synergistic effect that substantially increases the surface roughness of coal. Xu et al. [20] studied the wetting capacity of different structured anionic surfactants on coal dust by calculating the Hydrophile-Lipophile Balance (HLB) value. Their research indicated that the adsorption density depends on the hydrophobic interaction and electrostatic repulsion between surfactant molecules and coal dust. In dynamic wetting processes, surfactants with high HLB values can rapidly carry coal dust into the bulk solution. Zhang et al. [21] investigated the wetting characteristics of ionic liquids on coal through wetting experiments and energy spectrum experiments. They discovered that [C_12_MIm]Br ionic solutions can alter the ether bond functional groups on the coal dust surface, increasing its aliphatic hydrocarbon functional group content, thereby enhancing the wettability of coal dust.

Traditional experimental methods often face limitations in elucidating the underlying microscopic interaction mechanisms. In recent years, molecular simulation technology has emerged as a cutting-edge approach to bridge macroscopic properties with molecular structures, overcoming the constraints of traditional understanding and compensating for the shortcomings of experimental studies in revealing microscopic mechanisms. Zhang et al. [22] revealed the relationship between the dynamic wetting characteristics of zwitterionic surfactants on coal surfaces and the coal rank from a molecular dynamics perspective. Zhang et al. [23] improved dust suppression foam efficiency by formulating binary foam suppressants. They found that a blend of octylphenol ethoxylate (OP-5) and sodium dodecylbenzene sulfonate (SDBS) at a ratio of 12:24 exhibited optimal foaming and wetting properties, achieving maximum hydrogen bonding energy within the foam and interfacial interaction forces, resulting in excellent foam stability and wind erosion resistance. Ma et al. [24], using nanoindentation mechanical testing, studied the effects of SiO_2_ nanofluids and surfactants on microscopic mechanical parameters. They discovered that both could physically adsorb onto the coal surface, increasing hydrophilic sites, and thereby effectively reducing the mechanical strength of coal. Zhang et al. [25] macro- and microscopically verified the enhancing effect of surfactants on the wetting capability of halide salt inhibitors on bituminous coal. They identified ALSA as the most effective agent, successfully addressing the poor wettability, low moisture absorption, and short duration of action associated with traditional halide salt inhibitors. Liu et al. [26] through combined macro-experiments and micro-level molecular dynamics studies, found that 0.075 wt% DTAB exhibits the optimal wetting performance for coal dust in a weak acidic environment at pH = 3.

However, existing research has predominantly focused on macroscopic equilibrium parameters such as static contact angle and surface tension, making it difficult to reveal the kinetic characteristics of droplets during their actual interaction with bituminous coal surfaces. There is a lack of quantitative analysis regarding the dynamic wetting, penetration, and adsorption processes of droplets on bituminous coal. In practical applications, dynamic wetting ability determines the capture efficiency of dust suppression solutions for airborne floating dust. If only the static contact angle is considered while neglecting poor dynamic wetting performance caused by high surface tension, the dust suppression efficiency for hydrophobic coal dust will remain low. The primary purpose of adding surfactants is to reduce the solution’s surface tension and improve its dynamic wetting performance.

Bituminous coal holds the highest proportion of both reserves and production in China and globally. It generates substantial amounts of coal dust during mining, transportation, and utilization. Characterized by its high carbon content, low mineral matter and moisture content, and strong surface hydrophobicity, bituminous coal exhibits inherently poor natural wettability. Therefore, this study selects bituminous coal as the research subject. Three types of surfactants are mixed at different volume ratios. By integrating microscopic simulation with macroscopic experimentation, quantitative results on macroscopic wetting performance are obtained experimentally, while the observed phenomena are explained from a molecular scale via simulation. The dynamic wetting performance of surfactants with different hydrophilic active groups and their compounded solutions is investigated. This approach aims to determine the optimal concentration and composition of the compounded solution, reveal the synergistic mechanism of surfactant blending, provide crucial theoretical support for the development of high-efficiency dust suppression materials, and foster innovative applications in the field of coal mine dust control.

## 2. Materials and methods

### 2.1. Material selection and preparation

#### 2.1.1. Selection and preparation of coal samples.

The coal sample used in the experiment was a bituminous coal collected from the Huangyuchuan Coal Mine, Ordos City, Inner Mongolia Autonomous Region, China. The raw coal was pulverized using a ball mill and then sieved through a standard sieve to obtain coal dust with a particle size of 75 ~ 150 μm. The prepared sample was securely archived for future use. Proximate and ultimate analyses were conducted on the coal sample, and the results are presented in Table 1. M_ad_ represents the moisture content contained in the coal; A_ad_ denotes the ash content, which refers to the incombustible inorganic residue remaining after complete combustion of the coal; V_ad_ indicates the volatile matter, which represents the gases and vapors (excluding moisture) released when coal is heated to approximately 900 °C under anaerobic conditions; FC_ad_ stands for fixed carbon, referring to the solid combustible portion remaining in coal after the removal of moisture, ash, and volatile matter.

**Table 1:** Industrial element analysis of bituminous coal.

Proximate Analysis				Ultimate Analysis				
M_ad_	A_ad_	V_ad_	FC_ad_	C%	H%	N%	S%	O%
4.60	6.45	34.18	54.77	80.77	4.22	0.97	2.69	11.35

#### 2.1.2. Solution selection and preparation.

Due to the tendency of the polar groups in cationic surfactants to adsorb onto coal dust surfaces, their hydrophobic ends reduce the coal dust’s wettability upon contact with water. For this experiment, surfactants with wide availability, low cost, and effective wetting performance were selected, including: the anionic surfactant sodium dodecyl sulfate (SDS), the nonionic surfactant N,N-dihydroxyethyl cocamide (CDEA), and the zwitterionic surfactant cocamidopropyl betaine (CAB-35) [27].

**Fig 1:**
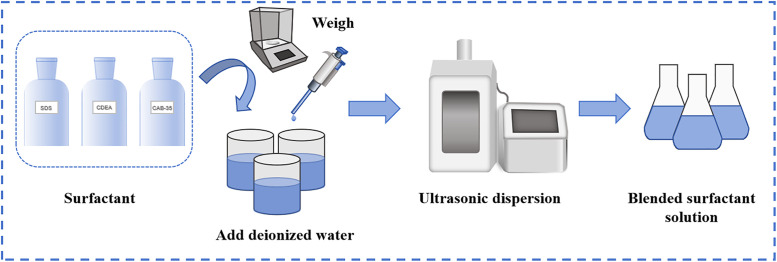
Experimental solution preparation.

Solutions were prepared at mass fractions of 0.01wt%, 0.03wt%, 0.05wt%, 0.07wt%, 0.1wt%, 0.2wt%, 0.3wt%, 0.4wt%, 0.5wt%, 0.6wt%, 0.7wt%, 0.8wt%, 0.9wt%, and 1.0wt%. The procedure for preparing the experimental solutions is illustrated in Fig 1.

### 2.2. Experimental and simulation methods

#### 2.2.1. Surface tension experiment.

The surface tension of each compound solution was accurately measured using an AEFS fully automatic surface tension meter with the platinum plate method. Prior to each measurement, the platinum plate was cleaned with ethanol and flamed over an alcohol burner to ensure a clean and contamination-free surface. During testing, the sample solution was maintained at 25°C in a constant-temperature water bath. Each sample was measured in triplicate, and the average value was taken to ensure data repeatability and reliability. The obtained surface tension data serve as the fundamental basis for subsequent analysis of wetting performance and synergistic effects.

#### 2.2.2. Contact angle measurement.

When a liquid droplet is placed on a solid surface, it may either spread spontaneously or form a specific contact angle with the substrate. In this study, contact angle measurements were carried out using an OCA25 video-based optical contact angle measuring system. Prior to measuring, the coal samples were pressed into smooth compact disks, and any loose dust on the surface was removed. During measurement, the droplet volume was controlled at 2.5 μL, and the ambient temperature was maintained at 25°C. Each sample was tested in triplicate, and the average value was recorded.

### 2.3. Simulation methods and parameters

#### 2.3.1. Construction of the bituminous coal molecular model.

To eliminate interference from mineral impurities in the bituminous coal samples on energy spectrum analysis results, this study employed an acid washing demineralization method for sample pretreatment. Specifically, 10 g of the bituminous coal sample was first mixed with dilute hydrochloric acid and magnetically stirred at 60°C for 6 h. Upon completion of the reaction, the sample was filtered and dried. Subsequently, the coal sample was treated with a mixed solution of hydrofluoric acid and concentrated hydrochloric acid under identical temperature and stirring conditions for another 6 h to effectively remove mineral impurities. The acid-treated coal sample was repeatedly washed with pure water and separated using a high-speed centrifuge until the supernatant reached neutrality. Finally, the resulting coal sample was dried in an oven to constant weight, yielding a high-purity, dried coal sample.

**Fig 2:**
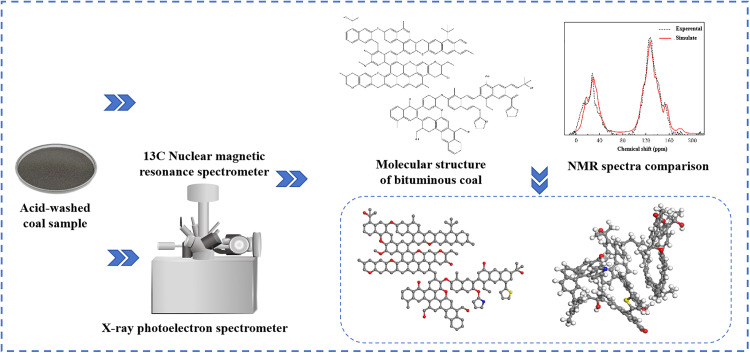
Construction of the bituminous coal molecular model.

The elemental composition of the coal sample, including C, H, O, N, and S, was determined by elemental analysis. The heteroatom structure and carbon skeleton structure of the coal sample were characterized using X-ray photoelectron spectroscopy (XPS) and 13C nuclear magnetic resonance (NMR) spectroscopy. Based on the comprehensive analytical results, the molecular structure of this bituminous coal was constructed. The rationality of the constructed coal molecule was verified using Mestrenova software. The process of constructing the bituminous coal molecule and the final molecular structure are shown in Fig 2.

#### 2.3.2. Molecular dynamics simulations.

**Fig 3:**
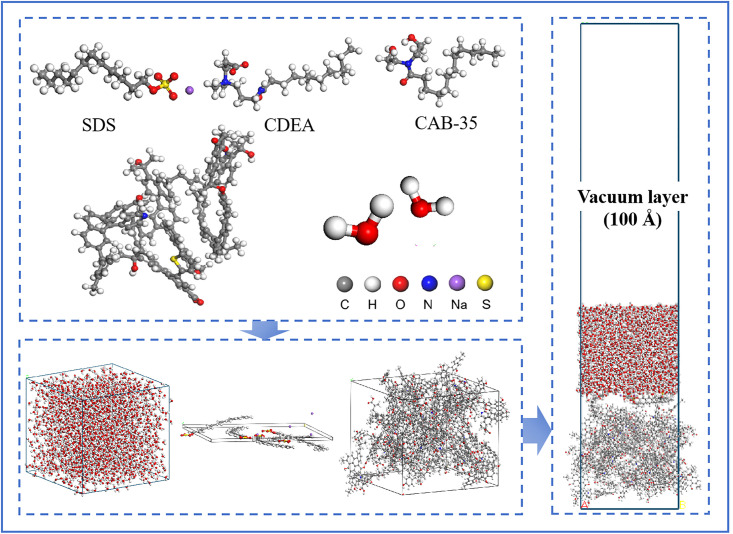
Construction of water/compound surfactant/bituminous coal model system.

Geometry optimization of the bituminous coal molecules and surfactant molecules was performed. An amorphous cell containing 15 coal molecules, 6 surfactant molecules, and 1500 water molecules was constructed with a base dimensions of 4 nm × 4 nm using the Amorphous Cell module. The Build Layer module was employed to establish the water/coal and water/surfactant/coal adsorption systems. A vacuum layer of 100 Å was added above the model to eliminate periodic boundary effects. The model was then geometrically optimized to achieve an energy-minimized stable configuration [28], as shown in Fig 3. The compounding parameters and identification codes of the surfactant molecules used in the molecular dynamics simulations are listed in Table 2.

**Table 2:** Surfactant molecular ratio and label in molecular dynamics simulation.

Surfactant molecular ratio	2:4	3:3	4:2
CDEA:CAB-35	A1	A2	A3
SDS:CAB-35	B1	B2	B3
SDS:CDEA	C1	C2	C3

The parameters for molecular system optimization were set as follows: Geometric optimization was performed using the Geometry Optimization task in the Forcite module, with the optimization accuracy set to Fine and the number of steps set to 50,000. The COMPASS II forcefield was selected, and charge assignment was set to Forcefield assigned. Annealing optimization was conducted using the Anneal task in the Forcite module, employing the NVT ensemble with the Nose thermostat method. The initial temperature was set to 300 K, and the maximum temperature was set to 600 K. The heating rate was 50 K per step, and the simulation time was 200 ps.

The parameters for molecular dynamics simulation were configured as follows: The Dynamic task in the Forcite module was selected, performed under the COMPASS II forcefield and NVT ensemble. The temperature was set to 298.0 K, controlled using the Nose method. Electrostatic and van der Waals interactions were calculated based on the Atom based method. The simulation time step was set to 1 fs, with a total simulation time of 500 ps. The calculation results from the production phase of the simulation were used for analysis. During this phase, system parameters such as energy, temperature, and pressure had reached equilibrium and stability. Sampling trajectories under this equilibrated condition are more representative of the true state of the system.

### 2.4. Permits statement

This study entailed material performance testing and numerical simulations, which did not involve human participants, animal experimentation, or the collection of any personal data. Therefore, in accordance with relevant national regulations and international norms, no specific ethical committee approval or permit was required. All experiments were conducted using standard equipment in the laboratories of Shaanxi Energy Institute.

## 3. Results and discussion

### 3.1. Analysis of bituminous coal wettability experiments

#### 3.1.1. Surface tension analysis.

**Fig 4:**
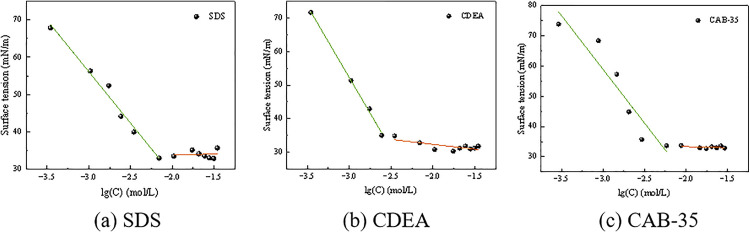
Surface tension of surfactant solutions at different concentrations.


The surface activity of a surfactant can be evaluated by its efficiency and effectiveness in reducing the surface tension of water [29]. The critical micelle concentration (CMC) is reached when a substantial amount of micelles appears in the surfactant solution. The corresponding surface tension at this point (γCMC) can be used to characterize the surfactant’s surface activity. The surface tension of surfactant solutions at different concentration gradients is shown in Fig 4. Furthermore, key surfactant parameters can be analyzed from this data, including the saturated adsorption amount at the gas-liquid interface ($\Gamma_{\max}$), the minimum area occupied per surfactant molecule at the interface ($A_{\min}^{s}$), the free energy of micellization ($\Delta G_{mic}^{0}$), and the standard free energy of adsorption ($\Delta G_{ads}^{0}$) [30,31]. See Eqs (1) to (4):

$$\Gamma_{\max}=-\frac{\text{1}}{2.303nRT}{\left(\frac{\partial \gamma }{\partial \log C}\right)}_{T}$$ (1)

$$A_{\min}^{s}=\frac{10^{16}}{N_{A}\Gamma_{\max}}$$ (2)

$$\Delta G_{mic}^{0}=nRT\ln\left(\frac{CMC}{55.5}\right)$$ (3)

$$\Delta G_{ads\;}^{\text{0}}=nRT\;\text{ln}\left(\frac{C_{\Pi }}{\text{5}\text{5}.\text{5}}\right)-\text{6}.\text{0}\text{2}\text{2}\Pi {\;A}_{m}^{s}$$ (4)

where, γ is the surface tension (mN/m); $\Gamma_{\max}$ is the saturated adsorption amount (μmol/m^2^); R is the gas constant, 8.314 J/(mol·K); T is the absolute temperature (K); C is the surfactant concentration (mol/L); ∂γ/∂logC is the absolute value of the slope of the linear segment preceding the inflection point in the surface tension curve; NA is the Avogadro constant, 6.02 × 10^23^; n is a constant with a value of 1.

As can be seen from Fig 4, the CMC and *γ*_CMC_ values for the three surfactants were obtained. Further calculations yielded the adsorption properties of the surfactants at the gas-liquid interface, presented in Table 3. The values of $\Delta G_{mic}^{0}$ and $\Delta G_{ads}^{0}$ for all three surfactants were negative, and |$\Delta G_{ads}^{0}$| > |$\Delta G_{mic}^{0}$|. This indicates that both the adsorption at the gas-liquid interface and the micellization process for these surfactant molecules are spontaneous, with a greater tendency for the molecules to enrich themselves at the gas-liquid interface via adsorption. The minimum area per molecule $A_{\min}^{s}$ at the critical micelle concentration reflects the spatial requirement for molecules to achieve maximally close packing at the interface. The order of this area, from largest to smallest, was SDS > CAB-35 > CDEA. This indicates that CDEA molecules form a tighter arrangement at the interface, while SDS molecules exhibit greater steric hindrance, resulting in a looser packing structure.

**Table 3:** Surface activity parameters of surfactant solutions.

Reagent	CMC		$\gamma_{CMC}$	$\Gamma_{max}$	*A*_*cmc*_	$\Delta G_{mic}^{0}$	$\Delta G_{ads}^{0}$
	%	mol/L	mN/m	mol/cm^2^	nm^2^/molecule	kJ/mol	kJ/mol
SDS	0.2	6.94E-03	32.98	4.83518E-07	3.44	−12.32	−28.39
CDEA	0.1	3.48E-03	34.86	7.47186E-07	2.22	−14.02	−32.30
CAB-35	0.1	2.92E-03	35.74	6.2411E-07	2.66	−14.46	−33.31

By measuring the surface tension values of the three surfactant monomers at different mass fractions, the critical micelle concentrations (CMC) for SDS, CDEA, and CAB-35 were determined to be 0.2%, 0.1%, and 0.1%, respectively. At their optimal concentrations, the three surfactants were blended pairwise. Mixed solutions were prepared at volume blending ratios of 1:1, 1:2, and 2:1, and their surface tension values were measured, as shown in Table 4.

**Table 4:** Surfactant compounding combination and surface tension.

Label	Surfactant compounding combination	surface tension (mN/m)
A1	0.1%CDEA:0.1%CAB-35 = 1:1	43.11
A2	0.1%CDEA:0.1%CAB-35 = 1:2	34.58
A3	0.1%CDEA:0.1%CAB-35 = 2:1	33.44
B1	0.2%SDS:0.1%CAB-35 = 1:1	32.76
B2	0.2%SDS:0.1%CAB-35 = 1:2	31.28
B3	0.2%SDS:0.1%CAB-35 = 2:1	36.54
C1	0.2%SDS:0.1%CDEA = 1:1	35.23
C2	0.2%SDS:0.1%CDEA = 1:2	33.71
C3	0.2%SDS:0.1%CDEA = 2:1	34.17

#### 3.1.2. Dynamic wettability analysis of compound solutions.

**Fig 5:**
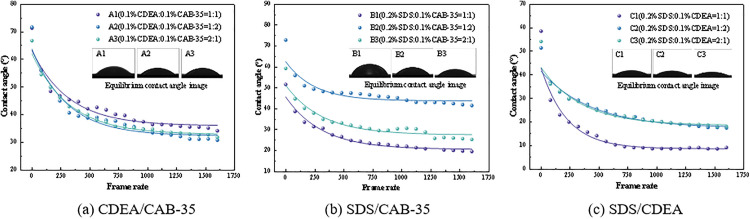
Dynamic contact angle change.

By measuring the contact angle of liquid droplets of equal volume during spreading and wetting on the interface, the rate of change of the contact angle reflects its spreading capacity over the interface. Assuming the attenuation rate of the spreading of the surfactant droplet on the interface is *K*, the contact angle at time t is *θ*, and attenuation rate of the contact angle over an infinitesimal time interval *dt* is **d*θ*, then the change in contact angle per unit time can be expressed as **d*θ*/*dt* = -**K*θ*. Integrating the above formula, and since the solid-liquid interface contact angle does not reach zero, a constant term must be added for correction. Therefore, the relationship between contact angle and time should satisfy the formula $\theta =\theta_{\varepsilon }+Ae^{-Kt}$, where A is the constant of integration and *θ*_*ε*_ is the equilibrium contact angle [31]. This study investigated the dynamic contact angle changes of blended solutions of three surfactants at their critical micelle concentration on a bituminous coal interface, as shown in Fig 5. The dynamic contact angle data, plotted against frame number, was fitted with an exponential decay function to derive the dynamic wetting parameters for each blended solution, presented in Table 5.

**Table 5:** Dynamic wetting parameters of solution.

Label	Fitted equation	*θ*_*ε*_	K
A1	y = 36.03 + 27.22*exp(-x/317.19)	36.03	0.00315
A2	y = 32.44 + 31.34*exp(-x/302.40)	32.44	0.00311
A3	y = 32.94 + 29.20*exp(-x/321.37)	32.94	0.00331
B1	y = 20.68 + 25.07*exp(-x/310.68)	20.68	0.00322
B2	y = 43.80 + 18.77*exp(-x/246.56)	43.80	0.00406
B3	y = 27.57 + 23.75*exp(-x/314.17)	27.57	0.00318
C1	y = 8.75 + 34.29*exp(-x/224.28)	8.75	0.00446
C2	y = 17.58 + 24.01*exp(-x/432.42)	17.58	0.00231
C3	y = 18.63 + 24.59*exp(-x/349.03)	18.63	0.00287

As can be seen from Table 5, the equilibrium contact angles, ranked from largest to smallest, are as follows: B2 > A1 > A3 > A2 > B3 > B1 > C3 > C2 > C1. The diffusion decay coefficients, ranked from largest to smallest, are: C1 > B2 > A3 > B1 > B3 > A1 > A2 > C3 > C2. Based on a comprehensive thermodynamic and kinetic evaluation, solution C1 (0.2% SDS: 0.1% CDEA = 1:1 volume ratio) exhibits superior spreading and wetting performance compared to the other blended solutions.

During the process of spray dust suppression, surfactant solutions achieve dust reduction by first adhering to coal dust particles, then spreading over the coal dust surface, and finally completely wetting the coal dust particles. The work of adhesion (*W*_*a*_) refers to the work required, or the corresponding decrease in system free energy, to convert a unit area of the liquid-vapor interface and solid-vapor interface into a solid-liquid interface when a liquid contacts a solid. It indicates the strength of interaction between the liquid and solid. A higher *W*_*a*_ value suggests stronger attractive forces between the solid and liquid, making it easier for the liquid to spread over the solid surface.

The spreading process involves the replacement of the solid-vapor interface by a solid-liquid interface, accompanied by an expansion of the liquid-vapor interface. The resulting decrease in system free energy per unit area is termed the spreading coefficient (*S*). This parameter reflects whether a liquid can spontaneously spread over a solid surface. When *S* > 0, the liquid spreads spontaneously, resulting in complete wetting of the solid. When *S* < 0, the liquid forms a finite contact angle, indicating only partial wetting.

The immersion process occurs when a solid is completely submerged in a liquid, causing the solid-vapor interface to be replaced by a solid-liquid interface, which lowers the system free energy. The magnitude of this energy reduction is generally referred to as the work of immersion (*W*_*i*_). *W*_*i*_ reflects the ease with which a liquid can completely wet a solid. A higher *W*_*i*_ indicates a stronger wetting ability of the liquid towards the solid, facilitating liquid penetration into solid pores or coverage of its surface [32,33]. According to $\gamma_{\mathrm{lg}}\cos\theta =\gamma_{sg}-\gamma_{sl}$ (Young’s equation), the calculation formulas are shown in Eqs (5) to (7).


$$W_{a}=\gamma_{lg}(1+\cos\theta )$$ (5)

$$S=\gamma_{\mathrm{lg}}(\cos\theta -1)$$ (6)

$$W_{i}=\gamma_{\mathrm{lg}}\cos\theta$$ (7)

where *γ*_sg_ is the solid-vapor interfacial tension (mN/m); *γ*_lg_ is the liquid-vapor interfacial tension (mN/m); *γ*_sl_ is the solid-liquid interfacial tension (mN/m).

**Fig 6:**
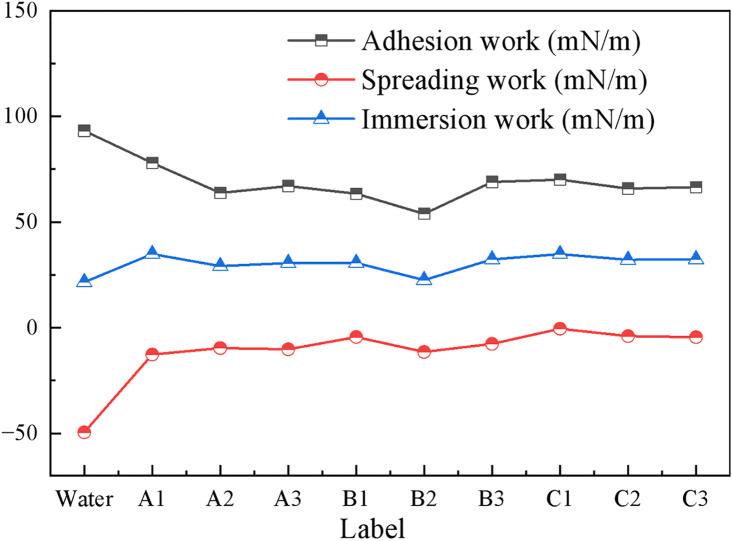
Adhesion work, spreading work and wetting work of the composite solution.

As shown in Fig 6, Pure water, solution A1, and solution C1 all exhibited relatively high work of adhesion. Among these, the significantly higher work of adhesion for pure water and solution A1 is primarily attributed to their higher surface tension. In contrast, among the solutions with relatively lower surface tension, solution C1 demonstrated the highest work of adhesion at 70.05 mN/m, indicating a stronger binding capability with the bituminous coal surface. Solution B2 showed the lowest work of adhesion, suggesting comparatively weaker binding ability. The spreading work (*S*) of all solutions is less than 0, indicating a tendency to contract into lens-like droplets on the bituminous coal surface rather than spread spontaneously. Pure water demonstrates the greatest spreading resistance at −49.43 mN/m, whereas solution C1, with a spreading work of −0.48 mN/m, is closest to meeting the condition for spontaneous spreading. The immersion work represents the energy required for the solution to wet the bituminous coal. Solution C1 exhibits an immersion work of 34.82 mN/m, which is higher than that of other blended solutions, indicating it more readily wets the coal.

In conclusion, the C group of blended solutions demonstrates superior performance. Among them, solution C1, characterized by high adhesion work, low spreading resistance, and high immersion capability, exhibits the most prominent dynamic wetting effect on the bituminous coal.

### 3.2. Quantum chemical simulation analysis

#### 3.2.1. Surface electrostatic potential analysis.

**Fig 7:**
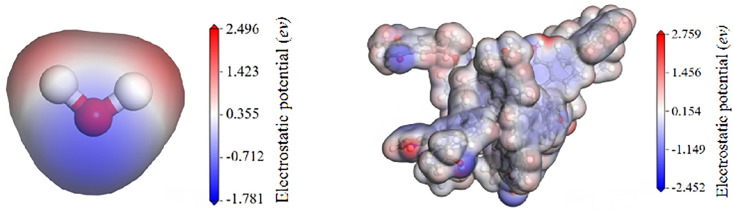
Surface electrostatic potential distribution of water and bituminous coal.

The surface electrostatic potential refers to the work required to move a unit positive charge from infinity to a specific point in the space surrounding a molecule [34]. Fig 7a shows the surface potential distribution of water. Calculations indicate that the maximum negative potential of a water molecule is −1.781 eV, and its maximum positive potential is 2.496 eV. By comparing the extreme values of the surface electrostatic potential of a substance with those of water, one can determine whether the substance is readily adsorbed by water. If the surface electrostatic potential of a substance is greater than that of water, it indicates that the substance can be adsorbed by water. As shown in Fig 7b, calculations for the bituminous coal molecule reveal a maximum negative potential of −2.452 eV and a maximum positive potential of 2.759 eV. Compared to the surface electrostatic potential of water, both the positive and negative potentials on the bituminous coal surface are greater. The regions of highest negative electrostatic potential on the bituminous coal molecule are primarily distributed around oxygen atoms and oxygen-containing functional groups. Therefore, water molecules are more readily attracted to the bituminous coal molecule, leading to the wetting phenomenon.

**Fig 8:**
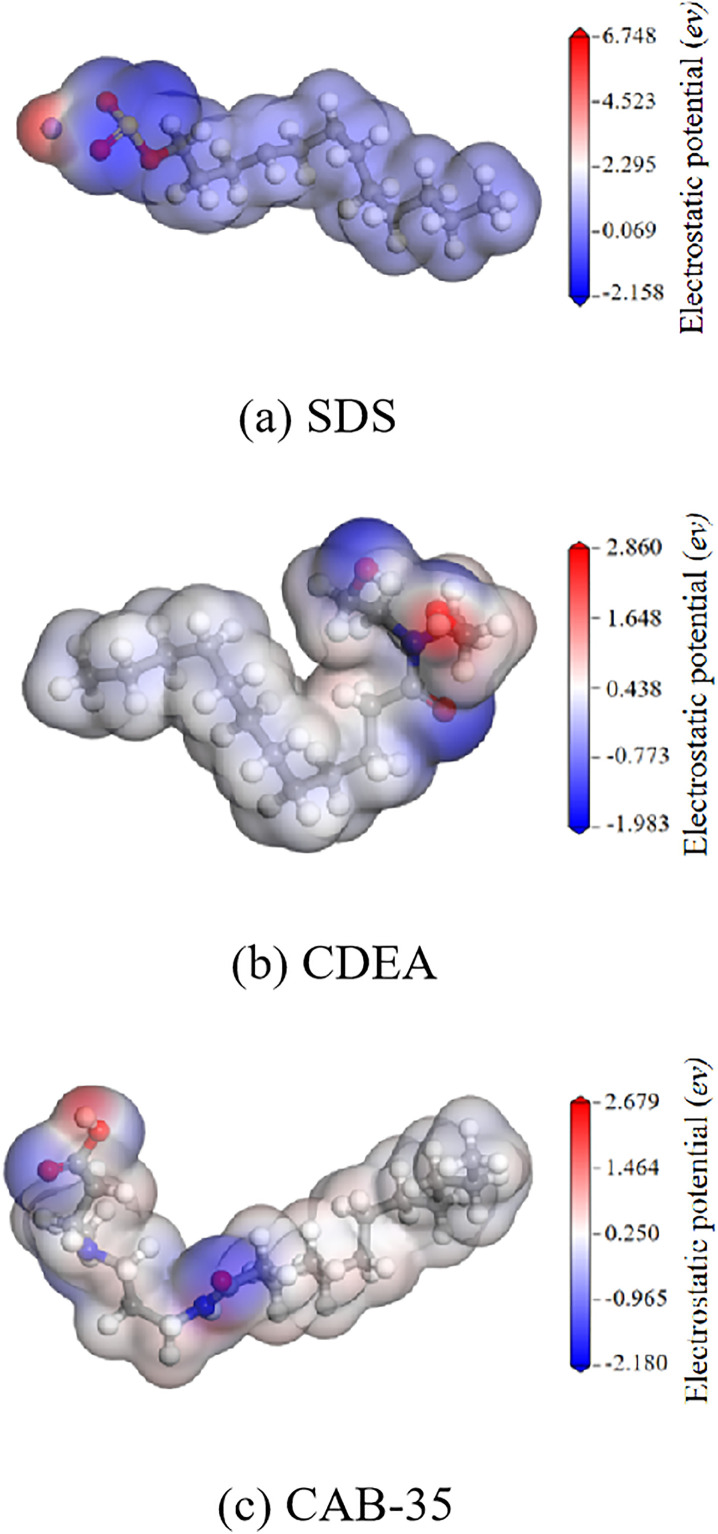
Surface electrostatic potential distribution of surfactant molecules.

As shown in Fig 8a–8c, the surface electrostatic potential distributions of SDS, CAB-35, and CDEA are presented. The extreme positive and negative potential values for SDS are 6.748 eV and −2.158 eV, respectively, with the extreme negative potential point located near the oxygen atoms of the sulfate head group. For CAB-35, the highest positive potential is 2.679 eV, located near the hydrogen atoms of the head group, while the extreme negative potential is −2.158 eV, situated at the carbonyl group of the head group. The CDEA molecule exhibits an extreme positive potential of 2.860 eV at the hydroxyl group in its head, and its maximum negative potential is −1.983 eV, located at the hydrogen atoms in the head group. The extreme values of both positive and negative potentials for all three surfactants are greater than the surface electrostatic potential of water. Therefore, it can be concluded that their head groups are hydrophilic, enabling adsorption with water molecules and thereby enhancing the wettability of bituminous coal. Since both the positive and negative potentials of CAB-35 and CDEA are located at their head groups, when acting at the coal-water interface, these head groups can adsorb both with water and with the surface negative potential sites of the bituminous coal molecules.

### 3.3. MD computational analysis

#### 3.3.1. Interaction energy analysis.

The solid-liquid interaction energy quantifies the strength of the interaction between the two phases and can serve as a parameter for evaluating the wettability of a solid surface [35]. The calculation is given by Equation (8):


$$E_{int(coal-water)}=\frac{E_{total}-E_{coal+surfacant}-E_{water}-E_{coal}-E_{surfacant+water}+E_{surfacant}}{\text{2}}$$ (8)

where, $E_{int(coal-water)}$ is the interaction energy between water and coal (kcal/mol); *E*_*total*_ is the total energy of the system (kcal/mol); *E*_*coal*_, *E*_*water*_, and *E*_*surfacant*_ are the energies of the coal, water, and surfactant, respectively (kcal/mol); *E*_*surfactant + water*_ is the total energy of the coal-surfactant system (kcal/mol); *E*_*coal* + *surfactant*_ is the total energy of the surfactant-water system (kcal/mol).

The interaction energies for different systems, calculated via molecular dynamics simulations, are presented in Tables 6 and 7.

**Table 6:** Interaction energy of single surfactant/water/coal system.

Reagent	H_2_O	SDS	CDEA	CAB-35
(kcal/mol)	−659.74	−670.05	−701.15	−626.23

**Table 7:** Interaction energy of composite surfactant/water/coal system.

Label	(kcal/mol)	Label	(kcal/mol)	Label	(kcal/mol)
A1	−681.01	B1	−637.29	C1	−705.80
A2	−697.55	B2	−678.72	C2	−664.34
A3	−643.70	B3	−586.24	C3	−700.38

As can be seen from Tables 6 and 7, the total interaction energies for all solution systems are negative, indicating that the adsorption process is spontaneous. The adsorption capacity between water and coal is further enhanced after the addition of surfactants, leading to improved interfacial stability. Among them, the C1 system exhibits a more significantly negative total interaction energy of −705.80 kcal/mol, indicating stronger interfacial binding between the bituminous coal and the solution, and consequently, better wetting performance. This phenomenon can be attributed to the formation of a robust, oriented hydrogen-bonding network between the negatively charged sulfate headgroups of anionic SDS and the polar amide groups of non-ionic CDEA, facilitated by electrostatic attraction. When only SDS is present at the interface, electrostatic repulsion between its like-charged sulfate headgroups prevents dense packing, resulting in significant molecular gaps. The incorporation of CDEA transforms this detrimental electrostatic repulsion into beneficial intermolecular attraction. Consequently, the electrostatic repulsion between headgroups is reduced, leading to tighter molecular packing at the interface and an increased interfacial adsorption density. Furthermore, the alkyl tail chain of SDS and the coconut oil chain of CDEA become entangled through van der Waals forces, further enhancing the mechanical stability of the interfacial molecular layer.

#### 3.3.2. Radial distribution function analysis.

The radial distribution function (RDF) can quantitatively characterize the differences in interaction between hydrophilic groups and the bituminous coal surface within a system, thereby revealing the underlying mechanism influencing wetting behavior. Its expression is given by Equation (9):


$$g(r)=\frac{\text{1}}{\rho }\cdot \frac{dN(r)}{4\pi r^{2}dr}$$ (9)

where, dN(r) is the number of atoms situated at a distance between *r* and *r* + *dr* from a central atom; *ρ* is the average number density of the atoms; 4π*r*2*dr* is the volume of the spherical shell of radius r and thickness.

**Fig 9:**
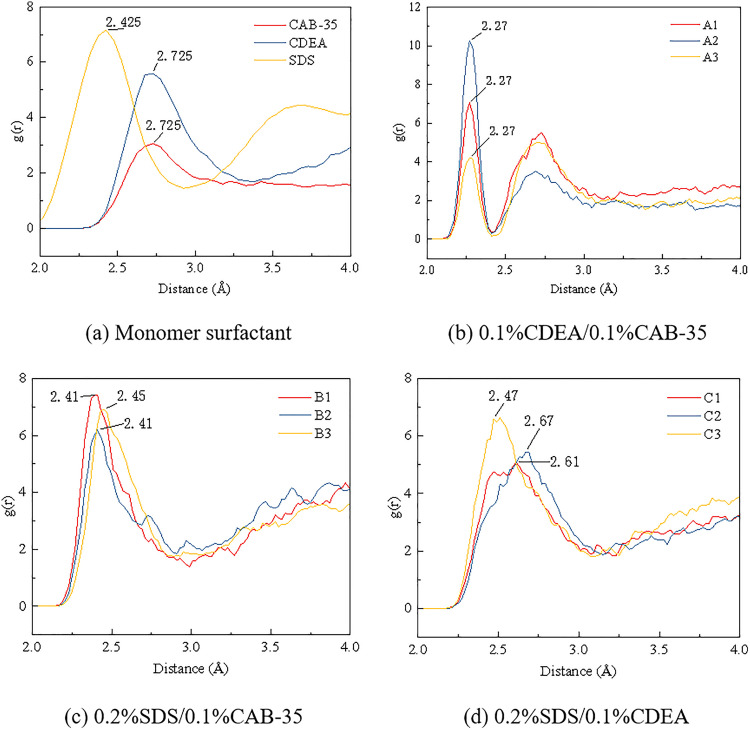
Radial distribution function of hydrophilic groups and water.

Fig 9 shows the radial distribution functions between hydrophilic groups and water in different systems. In Fig 9b, the RDF peak shifts to a lower distance, becomes higher and narrower. This indicates that the zwitterionic nature of CAB-35, which possesses both positive and negative charges, enables it to attract the hydroxyl groups of CDEA via electrostatic interactions, forming a complex. This reduces the electrostatic repulsion between the hydrophilic groups, leading to a more ordered arrangement of water molecules around them. In Fig 9c, the sulfate group of SDS and the quaternary ammonium group of CAB-35 form a tight ion pair, shortening the distance between them and causing the peak shift. Simultaneously, the carboxylate group of CAB-35 can partially neutralize the charge of the sulfate group, reducing the repulsion between the head groups and consequently decreasing the distance of the first hydration shell in the RDF. In Fig 9d, the hydroxyl groups of CDEA can form hydrogen bonds with the sulfate group of SDS. This hydrogen bonding interaction leads to a more orderly arrangement of the surfactants and lower intermolecular repulsion.

#### 3.3.3. Diffusion coefficient vector analysis.

The addition of different blended surfactants exerts varying influences on the motion of water molecules within the system. The migration behavior of water on the coal surface was analyzed based on the Mean Squared Displacement (MSD) curves of water and the self-diffusion coefficient D [36,37]. The expression for MSD is given by Equation (10):


$$MSD=N^{-1}\left(\sum {\mathrm{\backslash nolimits}}_{i}{\left|r_{i}(t)-r_{i}(0)\right|}^{2}\right)$$ (10)

where, N is the number of atoms; *r*_*i*_ *(*0*)* is the initial position vector of the i-th atom; *r*_*i*_ *(t)* is the position vector of the i-th atom at time t; The expression for the self-diffusion coefficient D is given by Equation (11):


$$D=\underset{t\to \infty }{\lim}\left(MSD/6t\right)=\frac{\text{1}}{\text{6}}K_{{}_{MSD}}$$ (11)

where *K*_*MSD*_ represents the slope of the MSD curve.

**Fig 10:**
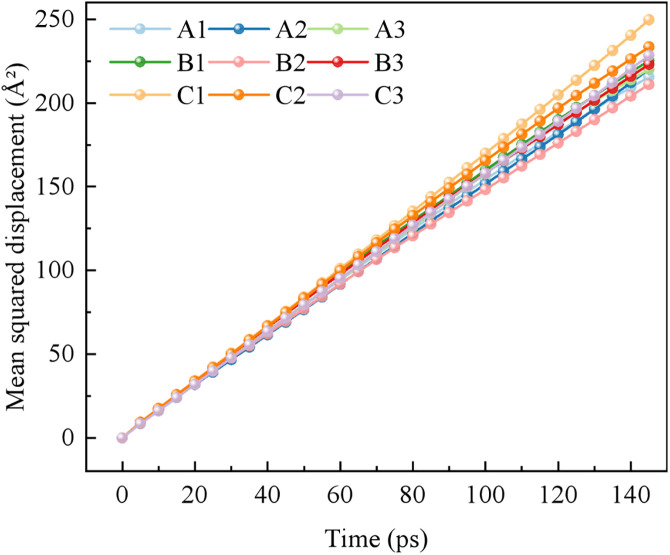
MSD curves of each system.

**Fig 11:**
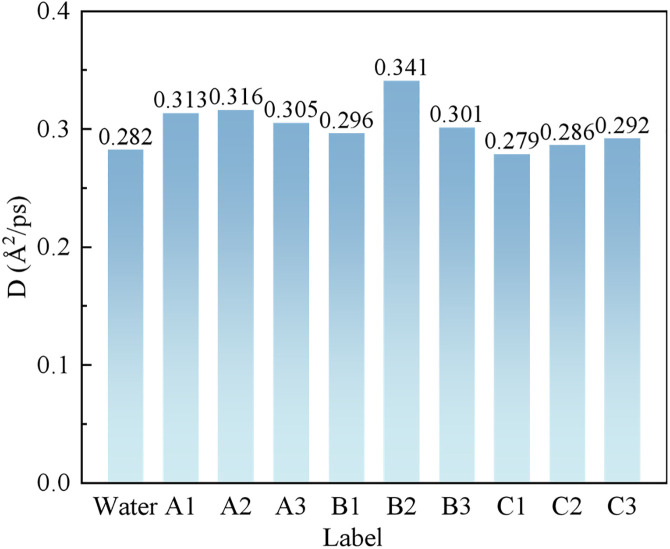
The self-diffusion coefficient D of each system.

The MSD curves and self-diffusion coefficients for the various blended systems are shown in Figs 10 and 11, respectively.

As shown in Figs 10 and 11, the self-diffusion coefficient (D) of water in the pure water system is 0.282. The addition of blended surfactants reduces the self-diffusion capacity of water molecules across all systems. The decrease in the D value is most pronounced in Group C, indicating a more restricted freedom of movement for the water molecules within these systems. This restriction is attributed to the electrostatic-polar interactions between the -OSO₃ ⁻ group of SDS and the head group of CDEA, which reduce the free diffusion of surfactant monomers, leading to a decrease in the D value. Additionally, the coconut oil-derived alkyl chains of CDEA may enhance inter-chain entanglement, further hindering diffusion.

### 3.4. Analysis of major functional groups in coal dust

**Fig 12:**
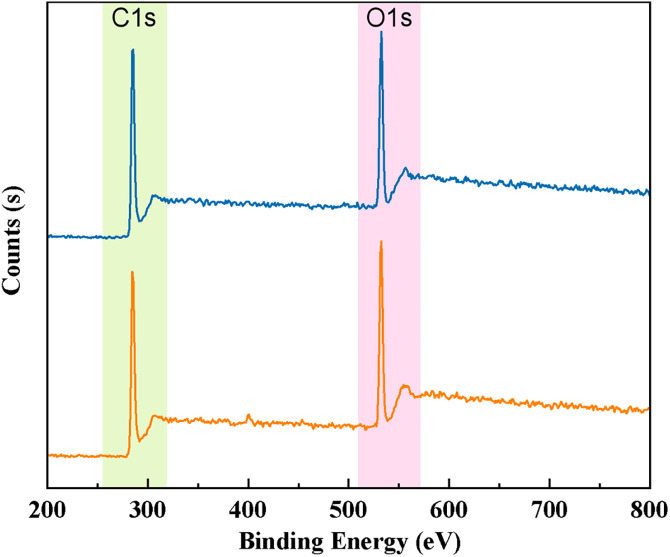
XPS spectra of coal samples before and after surfactant treatment.

Free radicals and chemical functional groups present in coal dust play a crucial role in studying the micro-scale bonding, chemical properties, and reaction behaviors of coal [38]. To investigate the effect of the blended solution on the functional group composition of coal dust, XPS analysis was performed on coal samples treated with pure water and the C1 blended surfactant solution, with particular focus on narrow-scan analysis of C and O atoms. Fig 12 and Table 8 present the semi-quantitative analysis of the content of elements such as C and O based on the wide-scan XPS spectra. After treatment with the blended surfactant solution, the C content on the coal surface decreased from 65.63% to 62.64%, while the O content increased from 25.43% to 25.9%. The content of other elements was low and thus not analyzed.

**Table 8:** The content of C, O and other elements on the surface of coal samples.

Sample	Element content (%)				
	C1s	O1s	Si2p	Al2p	N1s
Untreated bituminous coal	65.63	25.43	4.06	4.1	0.78
Treated bituminous coal	62.64	25.9	3.54	5.05	2.86

**Fig 13:**
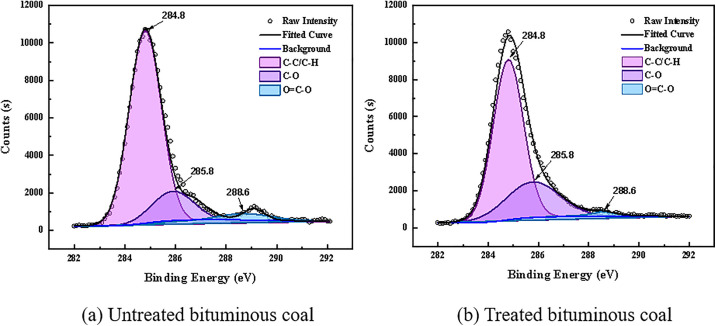
Results of the split peak fitting of C1s for coal samples.

**Fig 14:**
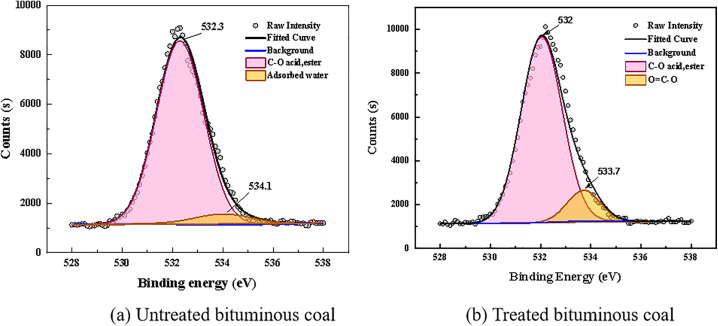
Results of the split peak fitting of O1s for coal samples.

The C1s XPS spectra of the bituminous coal samples before and after treatment are shown in Fig 13. After peak deconvolution, the spectra were resolved into three distinct peaks. The coal sample spectra exhibit prominent peaks at binding energies of 284.80 eV, 285.80 eV, and 288.60 eV, corresponding to functional groups of C-C/C-H, C-O, and O = C-O, respectively. As illustrated in Fig 13a, the C-C/C-H group is the most abundant carbon structure, accounting for 77.93% of the total. The contents of C-O (attributed to ether/hydroxyl groups) and O = C-O (assigned to carboxyl groups) account for 17.99% and 4.08%, respectively. In Fig 13b, after treatment, the content of C-C/C-H decreases to 61.97%, while the percentage of the oxygen-containing functional group C-O increases to 37.57%. This indicates that the surfactant broke the bridge bonds between aromatic rings in the coal sample, activating the C-H groups on the coal surface, which were subsequently oxidized to form a substantial amount of C-O groups. The C-C/C-H functional groups in raw coal primarily interact with air, which is unfavorable for water molecule adsorption. After being substituted by C-O groups, the number of hydrophobic sites decreases. This substitution enhances the interaction energy between the coal sample and water and reduces the interfacial free energy, thereby resulting in a decreased contact angle and improved wettability. To further investigate changes in the content of different oxygen-containing functional groups before and after treatment, a narrow scan of the O1s peak was performed. The fitting results are shown in Fig 14. The peak near 532.3 eV is assigned to C-O groups, and the peak near 533.4 eV is attributed to carboxyl groups (O = C-O). The peak observed at approximately 534.1 eV is attributed to O–H groups present in adsorbed water or within the oxidation layer. It can be observed that the content of oxygen-containing functional groups increased markedly in the coal sample after surfactant treatment.

### 3.5. Analysis of the surface morphology of coal dust

**Fig 15:**
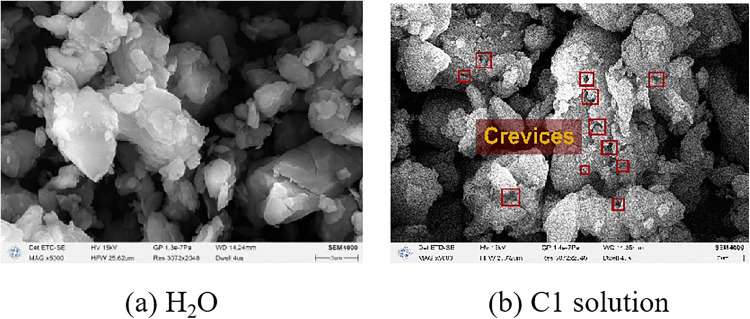
Surface morphology of bituminous coal after treatment.

The surface morphology of pulverized bituminous coal samples was examined using scanning electron microscopy. The Untreated bituminous coal surface appeared smooth and clean, with no visible adherence of inorganic minerals. After being ground for 40 min in a ball mill, coal powder with a particle size below 75 μm was obtained. Fig 15a shows that the fracture surfaces of larger particles remained relatively intact and smooth, with edges exhibiting distinct brittle fractures and numerous spherical and granular micro-attachments. As seen in Fig 15b, after immersion in surfactant solution, secondary pores and fractures developed on the coal sample surface due to chemical reactions. These fractures further facilitated the penetration of the solution into the coal matrix. The continuous expansion and propagation of these fractures significantly enhanced the wettability of the coal sample. This phenomenon occurs because surfactant molecules adsorb onto the oxygen-containing functional groups in bituminous coal through their polar head groups, weakening the strength of the carbon skeleton. The solution infiltrates the coal through newly formed pores, generating fluid pressure that promotes the expansion of micro-fractures into a interconnected pore network. The newly exposed pores on the bituminous coal surface reveal more polar sites, enhancing the dynamic wetting effect—a finding consistent with the experimental results presented in Section 3.1.2.

## 4. Conclusions

This study systematically investigated the dynamic wetting properties of surfactants with different hydrophilic active groups and their compound solutions through macroscopic experiments and molecular dynamics simulations. The optimal formulation was identified as the 1:1 volume ratio compound solution of 0.2% SDS and 0.1% CDEA, which exhibited superior dynamic wetting performance on bituminous coal. The synergistic mechanism between the two components was revealed, and the main conclusions are summarized as follows:

(1) Based on comprehensive thermodynamic and kinetic analyses, a robust, oriented hydrogen-bonding network forms between the sulfate groups of SDS and the polar amide groups of CDEA. The 1:1 volume ratio compound solution of 0.2% SDS and 0.1% CDEA demonstrates high work of adhesion and high work of immersion, indicating its propensity to remain on the coal surface and penetrate into the coal matrix. With a spreading coefficient of −0.48, approaching the condition for spontaneous spreading, the solution can actively cover the coal surface. This thermodynamically explains the excellent performance of this compound system observed in macroscopic experiments; its wetting behavior is “spontaneously driven” rather than forced by external energy.
(2) Molecular dynamics simulations further verified an interaction energy of −705.80 kcal/mol for the compound solution of 0.2% SDS and 0.1% CDEA formulated at a 1:1 volume ratio, which is a significantly more negative value. The surfactants form strong hydrogen bonds with water molecules, restricting the free movement of water near the hydrophilic groups. This reduces the local kinetic energy and entropy of water, promoting the ordering of water molecules and forming a low-mobility “interfacial bound water layer”. Together with the surfactants, this layer forms a stable hydration shell on the coal dust surface, which not only enhances initial wetting but also creates stronger capillary forces that bind coal dust particles more tightly, thereby improving dust suppression longevity.
(3) XPS and SEM analyses demonstrate that the compound solution fundamentally alters the chemical properties of the coal surface. After treatment, the proportion of hydrophobic C-C/C-H functional groups on the bituminous coal surface decreased from 77.93% to 61.97%, while oxygen-containing functional groups increased from 22.07% to 38.03%. The surfactants weaken the carbon skeleton strength of the bituminous coal, generating secondary pores and a fracture network that exposes more internal polar sites. This provides new pathways for further solution penetration and spreading, enhancing dynamic wetting effects and establishing a positive feedback cycle of “penetration-expansion-cracking-rewetting”. This self-reinforcing cycle continuously extends the depth and scope of wetting, ultimately disrupting and wetting the hydrophobic structure of the coal dust.
(4) Both SDS and CDEA are surfactants with mature production processes, readily available raw materials, and low cost. They exhibit good chemical and physical stability, ensuring long-term storage stability. Furthermore, both possess good biodegradability and are easily adsorbed and degraded in soil. However, SDS exhibits certain aquatic toxicity, making it more suitable for use in relatively enclosed mining areas. Strict wastewater management measures should be implemented to mitigate this potential environmental risk.
